# Project Tooth Fairy: a pan-London initiative from conception to delivery to patient-reported experience measures

**DOI:** 10.1038/s41415-023-5849-y

**Published:** 2023-05-26

**Authors:** Aisha Patel, Joe McQuillan, Joanna Johnson, Hope Sadio, Mohammed Dungarwalla

**Affiliations:** 057754072222738499628 Dental Core Trainee, Oral and Maxillofacial Surgery, Ashford and St Peter´s Hospitals NHS Trust, London Rd, Stanwell, Ashford, TW15 3AA, United Kingdom; 286208563145081395411 Clinical Director Dentistry, OMFS and Ophthalmology, Royal London Dental Hospital, Barts Health NHS Trust, Turner St, London, E1 1FR, United Kingdom; 162561807483584039014 Clinical Director of the Dental Directorate and Consultant in Paediatric Dentistry, Guy´s and St Thomas´ NHS Foundation Trust, Great Maze Pond, London, SE1 9RT, United Kingdom; 860614485304171367504 Senior Project Manager, North Thames Paediatric Network, United Kingdom; 311928137443311336045 Specialist in Oral Surgery, Department of Oral and Maxillofacial Surgery, Royal London Dental Hospital, Barts Health NHS Trust, Turner St, London, E1 1FR, United Kingdom

## Abstract

Pressure on paediatric dental general anaesthetic (GA) waiting lists has recently been at its highest, further compounded by the COVID-19 pandemic. Project Tooth Fairy (PTF), a pan-London collaborative project, was conceived in response to this backlog. A dedicated day case GA suite was established within The Royal London Dental Hospital (Barts Health NHS Trust) for use by multiple trusts to enhance elective recovery.

Over ten months, 895 patients were treated and discharged by PTF, averaging 101 patients per month. The majority required simple exodontia and comprehensive care and some patients were treated for surgery related to orthodontic treatment. Patient-reported experience measures highlighted an overall positive experience and appreciation for the service.

Several governance domains were considered in the service development, including risk management, workforce recruitment and information governance. Training opportunities have arisen for team members to develop their skills. Patient-reported experience measures have guided the provision of service focusing on paediatric dentistry and paediatric GA.

PTF has demonstrated the creation of a service centred around collaboration to successfully reduce GA waiting lists and therefore improving patient outcomes. The development of this service can be used as a template for the establishment of similar regional collaborative projects.

## Key points

- Following the COVID-19 pandemic, paediatric dental general anaesthetic waiting lists were in excess of 104 weeks, creating immense pressure on service providers.
- A pan-London collaborative project to reduce dental paediatric general anaesthetic waiting times by creating of a new general anaesthetic treatment suite at the Royal London Dental Hospital began.
- As a result of Project Tooth Fairy, waiting times have successfully been reduced and continue to do so. Unique opportunities for collaboration, training and career development have been created.

## Introduction

Dental caries remains among the most prevalent childhood disease in England and is the most common reason for hospital admission among 6-10-year-olds.^1^ In 2019, Public Health England reported that 23.4% of five-year-old children in England experienced dental decay, and the proportion of teeth with dental caries requiring extraction was 10.7%.^2^ Before the COVID-19 pandemic, tooth extraction was the predominant procedure carried out on 5-9-year-olds in hospitals,^3^ and during 2018-2019 alone, there were 59,011 finished consultant episodes for 0-19-year-olds admitted for dental extractions.^1^

If left untreated, the burden of dental caries can be vast, impacting general wellbeing, mental health and development. The *Child dental health survey*^4^ published in 2015 reported that 58% of 12-year-olds and 45% of 15-year-olds felt their daily lives were impacted by their oral health due to difficulty eating, speaking, sleepless nights, and being too embarrassed to smile or laugh. On average, children experiencing toothache and dental infection are likely to miss three school days, affecting their education and social development. Furthermore, advanced tooth decay will lead to dental sepsis, with 1% of five-year-olds affected by the life-threatening condition.^5^

The ongoing COVID-19 pandemic has had unprecedented effects on the provision of dental care. Nationally in 2019/20, 35,190 dental extractions were performed on children aged 0-19; however, this fell drastically to less than half in 2020/21, with 14,645 extractions performed.^1^ This decline is attributed to the challenges in providing services compared to pre-pandemic rather than a lack of need, as the incidence of dental caries among five-year-olds in London is just over 25%, higher than the national average.^6^ During the height of the pandemic, all elective paediatric dental surgery under general anaesthesia (GA) ceased across London, restricting care to emergency and urgent cases only. Consequently, once elective surgery was resumed in March 2021, there was extreme pressure, with a waiting time of over 102 weeks. The paediatric GA waiting list increased by 61%, from approximately 2,500 children waiting in March 2020 to approximately 4,000 in 2021 (from unpublished local data). As already highlighted, the backlog of patients waiting for dental extractions results in thousands of children suffering from pain and infection.^7^ Following the peak of the pandemic, recovery is slowly taking place nationally, with 31,968 extractions performed on 0-19-year-olds in the 2021/22 period.^8^

In response to the growing paediatric GA wait times across London, NHS England commissioned a pan-London collaborative solution called 'Project Tooth Fairy' (PTF). PTF aims to reduce the extensive waiting list of children awaiting GA for minor dental surgery by creating a high-volume, low-complexity hub, consisting of three dental procedure rooms at the Royal London Dental Hospital (RLDH) under Barts Health NHS Trust (BHNT). Funding was provided by NHS England collaboratively with the North Thames Paediatric Network. PTF receives a referral from six participating hospitals and three community dental services: Barts Health Trust; Guy's and St Thomas' Trust; University College London Hospital; Kings College Hospital London; Whittington Hospital; Chelsea and Westminster Hospital; Kent Community Dental Services; Kings Community Dental Services; and Bromley, Greenwich and Lewisham Community Dental Services.

Patients are treatment planned by specialist paediatric dentists within their trusts and referred to the project for treatment by the same trust's team or an external team where agreed. Procedures completed include comprehensive care (including restorations, stainless steel crowns, endodontics and basic periodontal care and routine and surgical exodontia).

The project aims to run until March 2023, after which the area will be repurposed for use by dental specialities within Barts Health NHS Trust. The service commenced on 4 October 2021 and was forecast to treat 290 children per week, thus aiming to reduce the pan-London backlog to below an 18-week waiting time by the summer of 2022.

## The local picture

The Royal London Dental Hospital is situated within the borough of Tower Hamlets. Tower Hamlets possesses the highest child poverty rate of all the London boroughs and in the UK, with 51% of children living in a household experiencing poverty compared to 35% in the typical London borough.^9^^,^^10^ The stark difference in poverty rates between Tower Hamlets and other London boroughs is also reflected by the 39.8% prevalence of dental decay experience in Tower Hamlets, which is considerably higher than the prevalence for London and England (27% and 23.4%, respectively).^2^ Another major contributory factor in the prevalence of dental caries is ethnicity. Tower Hamlets is home to the largest Bangladeshi population in England, with 34.6% of residents being of this ethnicity.^11^ The 2019 *Child dental health survey* reported that the Bangladeshi population had approximately 38% caries prevalence among five-year-olds.^2^ These figures highlight the local population's significant need for dental services.

## Service development

The unique collaborative pan-London nature of PTF created unique challenges in terms of infrastructure, information governance, workforce and risk management.

### Converting a dental clinic into procedure rooms

The initial proposal to create theatre space for PTF was to install a modular theatre block in the RLDH car park. However, a more cost-effective option of transforming existing clinical space into an outpatient GA suite was suggested. The suite consists of an admission room, three procedure rooms, three anaesthetic rooms, a recovery area and a step-down facility. The existing activity was relocated to available chairs across the dental hospital network.

New operating theatres in the UK require between 20-25 air changes per hour and for the theatre to be maintained at positive pressure to surrounding areas to reduce the risk of surgical site infection. An air change is defined as the air volume of the room being supplied to or removed from the room.^12^^,^^13^ In comparison, treatment rooms used for minor surgical procedures using anaesthetic gases should have a minimum of 15 air changes per hour.^14^ The PTF treatment rooms achieve this, to also align with new infection control policy developed due to COVID-19.^15^

### Treatment planning

Patients referred to PTF are assessed and planned for GA treatment by specialist paediatric dentists within their own trust, who have skill and experience in childhood caries management to ensure treatment plans are quality assured. A precise treatment plan is established between the clinician, patient and their parent/guardian. At this point, it would be confirmed whether the patient meets the criteria for PTF. The treatment plan is then documented on a dedicated PTF referral form, along with a copy of the patient's notes (including relevant radiographs) and a copy of the consent form from the external trust.

The downside to external treatment planning and clerking is the reliance on the external team, who may not be readily available to discuss queries at short notice. This has been overcome by ensuring only named clinicians at external sites are able to refer to PTF, who are quickly accessible by email and telephone.

### Information governance

Patients from within BHNT have their notes saved electronically, readily accessible by clinicians who have password-protected access to the online patient record system. Consent forms remain paper-based and are kept in a hard-copy file, which is transported to the GA suite on the day of surgery.

### Workforce management

The project has utilised and created dedicated roles for dentists (including specialists in paediatric dentistry and oral surgery); anaesthetists; anaesthetic assistants; dental nurses; dental matrons; play specialists; receptionists; schedulers; service managers; theatre scrub teams; operating department practitioners (ODPs); cleaning and maintenance staff; and porters.

Specific posts for paediatric dental specialists and specialist oral surgeons were created to lead in the delivery of treatment, utilising dentists at the top of their scope of practice. In addition, clinicians were recruited from community dental services who previously did not have access to GA lists, allowing further professional development, increased continuity of care to their patients and increased job satisfaction.

Paediatric anaesthetists were recruited to work alongside general anaesthetists, providing a valuable opportunity for upskilling and diversifying.

### Prioritisation of patient list

Another challenge was the organisation of the patient list. NHS England commissioners stated that the longest waiters across London would be prioritised and treated first. On assessment of the patient list across London, the vast majority of the longest waiting patients were within the North East London (NEL) waiting lists. Therefore, in discussion with all providers and the managed clinical networks, a decision was made to treat BHNT and NEL patients only for the first eight weeks to ensure those patients who had waited for the longest were treated first. This agreement was a testament to the collaboration between the providers to stop historical thinking about 'their own' patients and adopt a pan-London ethos. Once the longest waiters across NEL were in line with the other integrated care system areas, the facility was opened to referrals from other providers to ensure both activities were maintained and waiting lists across London reduced in tandem.

### Clinical pathway

PTF accepts referrals for paediatric day cases of patients aged 2-16 without complex co-morbidities and where their diagnoses meet the eligibility criteria set out.

The exclusion criteria include: children under two years of age; weight less than 12 kg; sleep apnoea; pre-term infants of 32 weeks gestation; and a family history of sudden infant death syndrome. Appendix 1 shows the exhaustive inclusion and exclusion criteria.

A referral is made by completing an online proforma which is securely sent electronically to BHNT. Once referred to PTF, patients are allocated to an exodontia-only list, complex oral surgery (expose and bond and removal of impacted or supernumerary teeth) or comprehensive care list (full mouth rehabilitation with extractions, restorations, crowns).

**Fig. 1:**
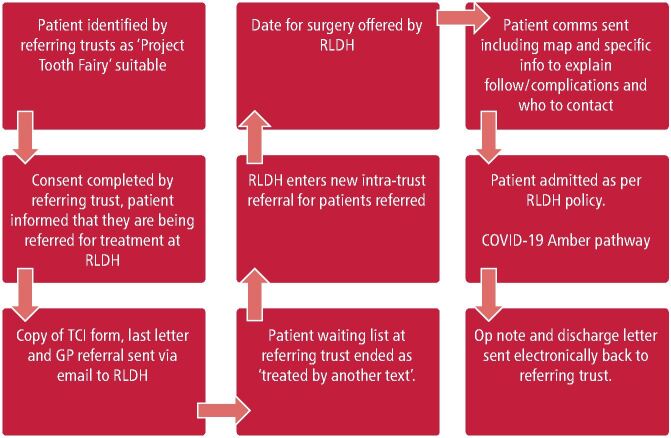
The clinical flow of patients who are referred to PTF

It has been considered that this acceptance criteria may increase inequalities; however, this is a limitation of any day case unit, as safety must take precedence. Although the acceptance criteria are strict, it has enabled routine patients to be treated through the new facility, which consequently has created space for more complex patients to be seen by consultants within their own trust. Additionally, as the project has progressed, the acceptance criteria has slowly increased to include children with additional needs, such as autism. Figure 1 shows the clinical flow of patients who are referred to PTF.

On the day of admission, the child and parent/guardian will check-in at the reception and are then seen in purpose-built consultation rooms. A nurse will assess for starvation instruction adherence, height, weight and general clerking. Next, the anaesthetist will assess the patient and discuss the mode of anaesthesia with both the patient and parent/guardian. There is the possibility of gas induction or conventional intravenous induction. The anaesthetist will also decide if pre-medication is required, and if so, this is later administered in the anaesthetic room. The dental team will also assess the patient and discuss the planned procedure. The consent completed by the referring trust or community dental service is confirmed by the anaesthetist and two dental clinicians confirm the teeth to be treated and the general surgical risks involved. A play specialist will meet each patient using props to demonstrate cannulas, face masks and other anaesthetic devices.

As per the World Health Organisation convention for safe surgery, after clerking all patients for that session, there is a team brief, at which point any deviations to the treatment plan and list order are discussed and managed. A sign-in procedure is completed in the procedure room with an ODP and anaesthetist. Routinely, the patient is anaesthetised in the procedure room with their parent/guardian and a play specialist. Once the patient has been anaesthetised, the World Health Organisation surgical safety checklist is completed to confirm the child's consent and details.^16^

**Fig. 2:**
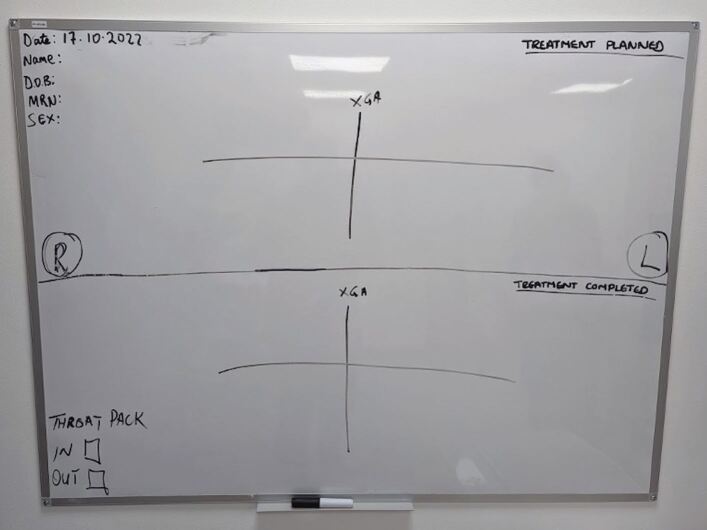
A whiteboard used in all procedure rooms. Teeth to be extracted are called out during 'sign in' and the teeth extracted are called out by the operator and written on the board by a General Dental Council-registered professional. All team members who work in the PTF rooms conform to this methodology, which is introduced during their induction

The treatment plan is verified from the consent form and written on a whiteboard in the procedure room. A two-person countdown is performed to minimise the risk of wrong-site surgery. Both operator and assistant confirm the tooth to be extracted before any instruments are applied to the tooth.^17^ The operating surgeon calls out any teeth extracted, confirmed by a second person, and the tooth is marked on the whiteboard (Fig. 2).

Once the procedure is complete, the teeth extracted are again verified against what is written on the whiteboard to ensure the exact procedure was performed, and any discrepancies are discussed. The patient is then discharged to a recovery ward for same-day discharge. Once the child is awake, the parent and child are taken to the discharge lounge. Before discharge, the patient is deemed safe by the anaesthetist and is also reviewed by the treating clinician.

**Fig. 3:**
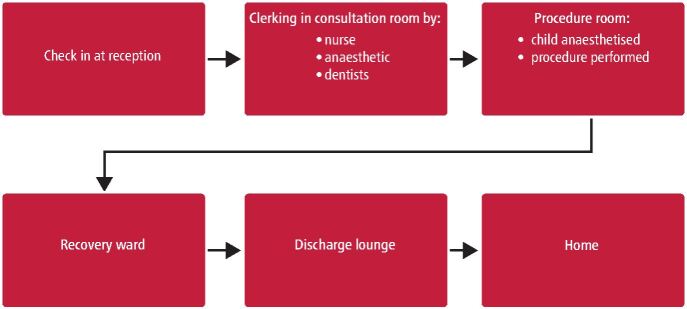
The flow of patients on the day of their procedure in PTF

The operation notes and discharge summary are recorded on the secure online patient record system and are sent back to the referring trust electronically via secure NHS.net email (Fig. 3).

### Risk management

In a medical emergency, patients are treated as per the Resuscitation Council guidelines. As such, all staff must keep up to date with medical emergency training and paediatric life support. Should the situation escalate and require emergency medical attention, staff must follow BHNT policy to make an internal crash call, prompting an emergency team to arrive within three minutes. If the patient needs to return to the theatre, the responsible surgical consultant, on-call paediatric anaesthetist and theatre team must be informed.

### Training opportunities

Although training was not an initial aim of PTF, once the service was established and running, an opportunity for this arose. It was estimated that each procedure room would run two lists per day, with six patients per list for six days a week, giving 36 lists per week and up to 216 patients seen. The activity level provides ample training opportunities for theatre staff, anaesthetists and dentists to develop competencies in their respective fields. Specifically for dental undergraduates, dental core trainees and speciality registrars, the opportunity to gain one-to-one teaching under GA is invaluable. It enables skills and techniques to be developed without the usual behaviour and patient management needed for treatment under local anaesthetic or conscious sedation.

## Quantitative and qualitative outcomes

Data were collected prospectively by PTF administrators from October 2021, when PTF first started treating patients, to July 2022, the time of writing this publication. The number of patients discharged per month, their referring trust and the type of care provided was recorded in a Microsoft Excel spreadsheet. All data were anonymised and stored electronically on a secure network.

All parents and carers of children treated by PTF were asked to complete an anonymous paper-based questionnaire to gain feedback on the service. These responses were collated and organised by the score given.

**Fig. 4:**
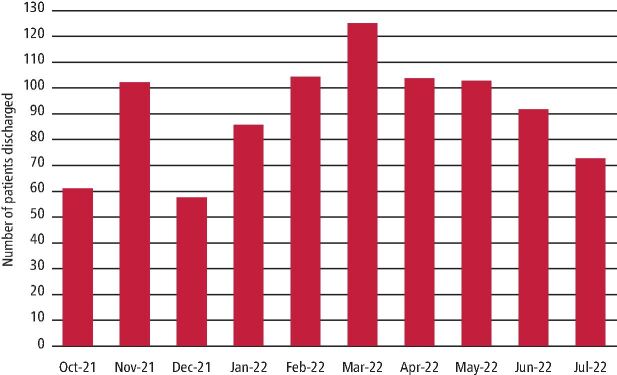
Number of patients seen in PTF between October 2021 and July 2022

From October 2021 to July 2022, 895 patients were treated and discharged by PTF, averaging 101 patients per month (Fig. 4). The month with the lowest activity was December 2021, treating only 57 patients. This was likely due to a high level of staff and patient illness due to the Omicron COVID-19 variant and annual leave around the Christmas and New Year period. The month with the highest activity was March, treating 125 patients.

**Fig. 5:**
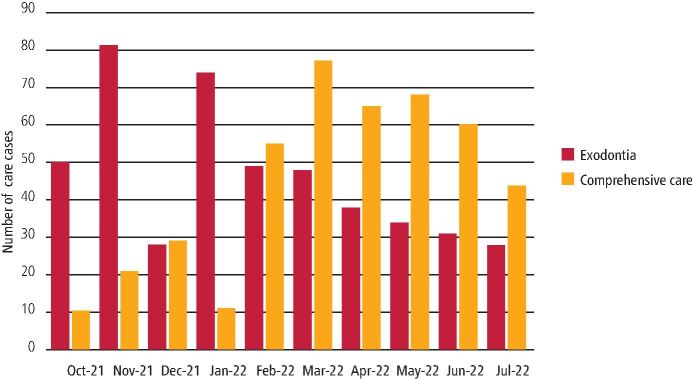
The mix of comprehensive care and exodontia cases between October 2021 and July 2022

During the initial months of PTF, exodontia cases were treated in a much higher proportion than comprehensive care. However, from February 2022 to June 2022, a more significant proportion of comprehensive care cases were seen (Fig. 5).

**Fig. 6:**
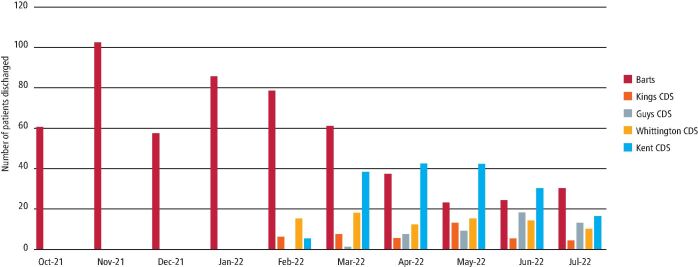
The trusts from which patients were treated on PTF from October 2021 to July 2022

PTF initially treated patients from within BHNT. Patients from external referrals were first seen in February 2022. Following BHNT, most patients were referred from Kent Community Dental Services (Fig. 6).

The development of PTF proposed several challenges to be overcome, and as with any new service, there are still flaws to be addressed. At its conception, PTF was forecasted to treat 290 children per week. Our analysis of patients discharged to date showed that, on average, 102 children are treated per week; less than half the prediction. The reduced capacity could be due to several factors. Firstly, many patients seen were long waiters; therefore, they had to be re-assessed and their treatment plans re-verified before appointing a surgery date. Hence, some clinical capacity was dedicated to revalidating the long waiting patients, taking away treatment time.

Before the establishment of PTF, the wait time for dental paediatric GA care across London was more than 104 weeks. This has reduced to no patients waiting longer than 78 weeks. Within BHNT, the comprehensive care list was more than 40 weeks. However, now the wait times for both comprehensive care and exodontia lists have been reduced to 12 weeks or less, demonstrating the impact this service has had on ensuring children receive the care they urgently need.

### Feedback: patient-reported experience measures

Gaining feedback from the patients and parents/carers treated by PTF is vital to understanding how well the service meets the needs and expectations of the population it aims to serve. Data on patient-reported outcome measures and patient-reported experience measures have already demonstrated reliability in determining how effectively a service meets patients' needs.^18^

Upon discharge, parents/carers are asked to complete a short questionnaire to rank the service in various aspects using a 10-point Likert scale, with ten being very positive/very well and zero being very negative/not well at all. There was also the option to leave other comments. At the time of writing, a total of 57 responses had been collected and analysed. Overall, the responses gained are positive, with no scores below seven in any question (Appendix 2).

### Beyond Project Tooth Fairy

Currently, adult special care dentistry and oral surgery patients who require anaesthetist-led sedation or GA are seen in the Royal London Hospital main theatres. The main theatres are shared across all medical specialities, including emergency and elective cases, hence waiting times for these patients can be lengthy, as dental cases may be considered low priority. Having rooms with the capacity to deliver GA cases within the dental hospital would enable these patients to be treated much sooner, allowing the trust to meet waiting list targets. BHNT are looking to introduce adult GA oral surgery operations to the PTF theatres as the project comes to an end.

## Conclusion

PTF has successfully demonstrated that collaborative projects can deliver care effectively and safely in response to exponential demands placed on all trusts, particularly as a result of the COVID-19 pandemic.

The authors have personally benefited from being able to experience teaching opportunities, working with colleagues from external trusts thus bringing a varied skill mix, one-to-one teaching opportunities, opportunities to teach colleagues in allied specialities, becoming involved in quality improvement projects, and broadening surgical experiences, which can only benefit our patient cohort in the long-term.

**Figure:**
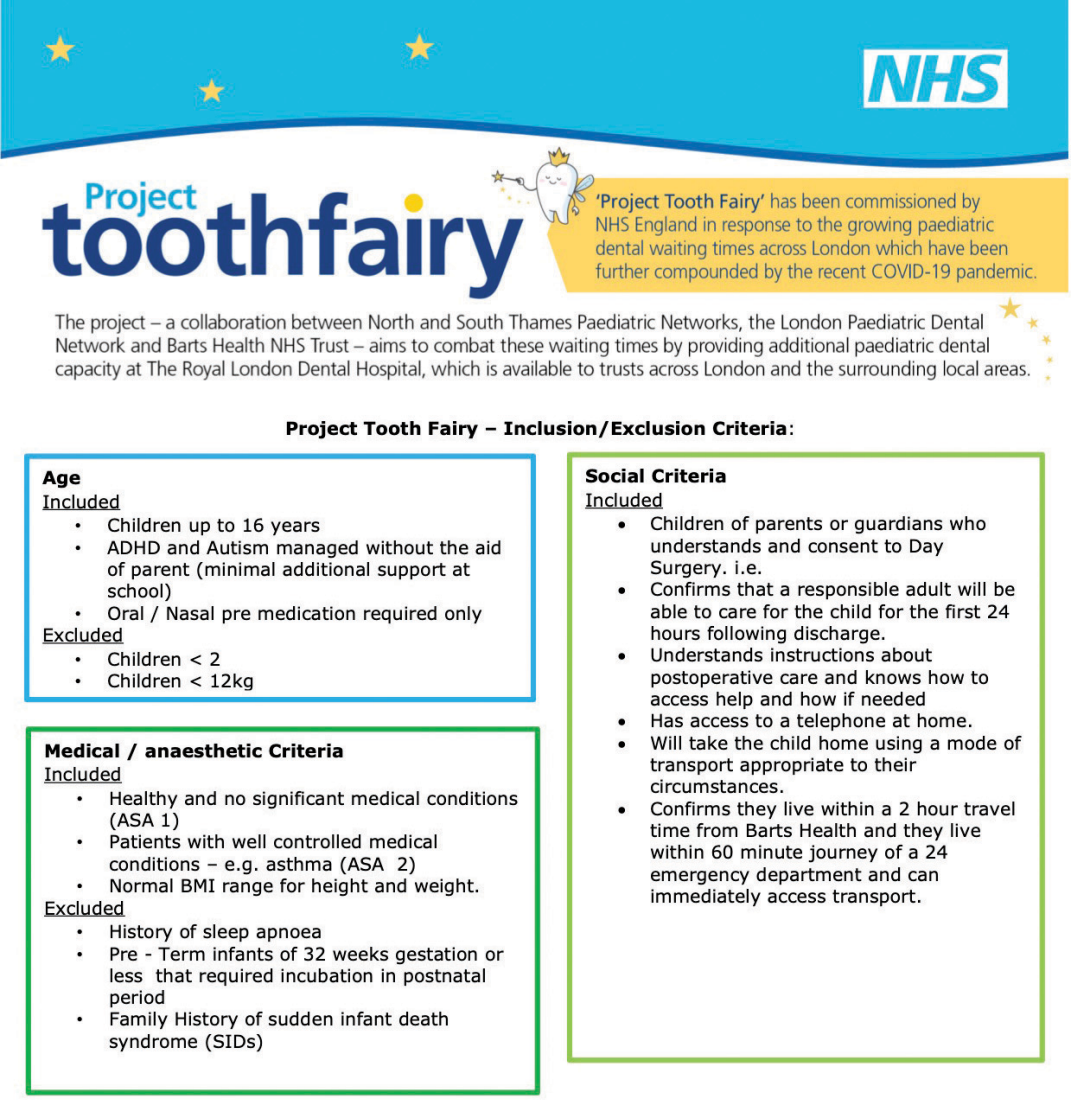
Appendix 1 Criteria for patients being referred to PTF

**Figure:**
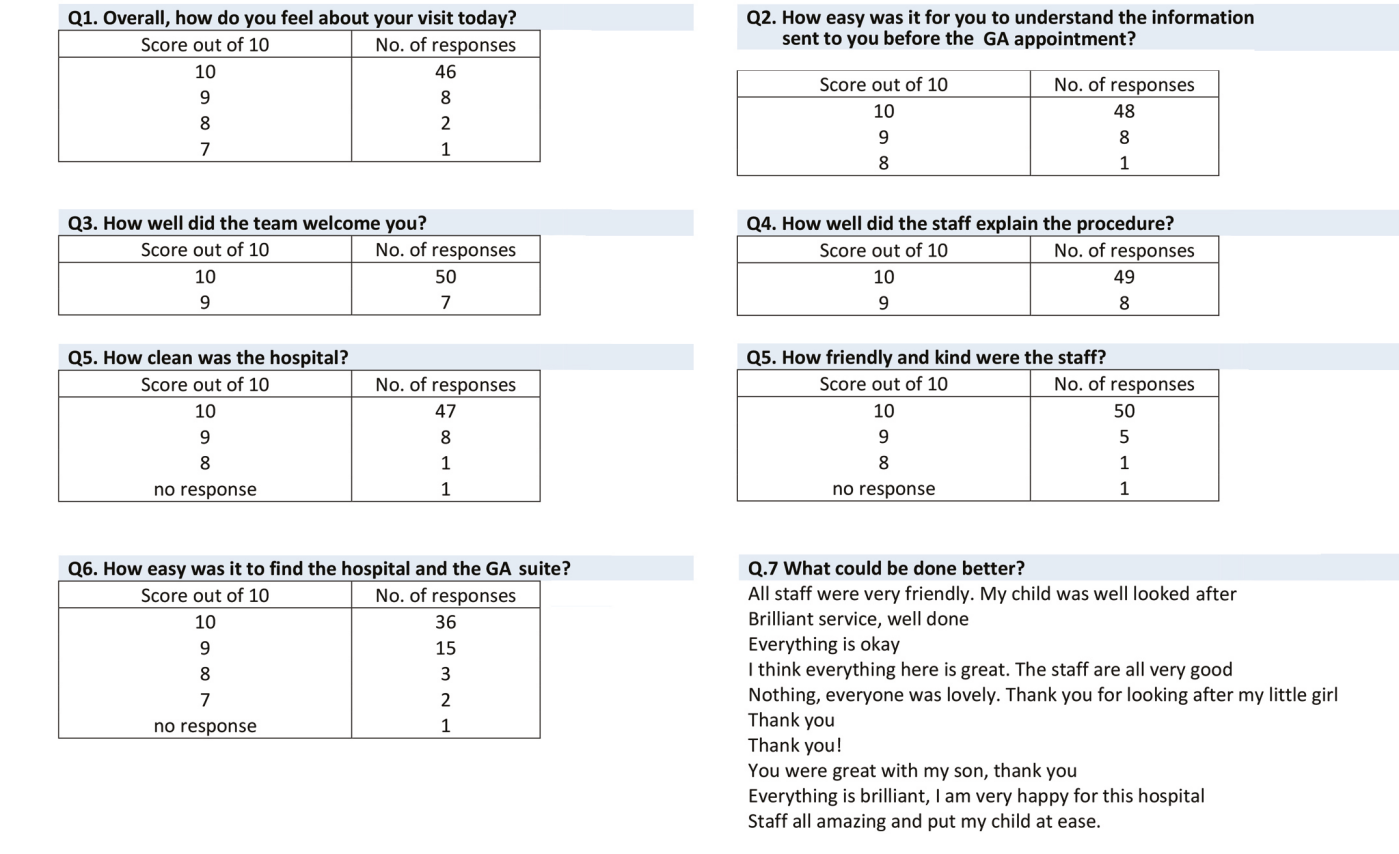
Appendix 2 Feedback obtained from parents/carers of children treated by PTF
